# The Human Phospholipase B-II Precursor (HPLBII-P) in Urine as a Novel Biomarker of Increased Glomerular Production or Permeability in Diabetes Mellitus?

**DOI:** 10.3390/jcm13092629

**Published:** 2024-04-30

**Authors:** Shengyuan Xu, Anders Larsson, Lars Lind, Cecilia Lindskog, Johan Ärnlöv, Per Venge

**Affiliations:** 1 Department of Medical Sciences, Clinical Chemistry, Uppsala University, SE-751 85 Uppsala, Sweden; shengyuan.xu@medsci.uu.se (S.X.); anders.larsson@akademiska.se (A.L.); 2 Department of Medical Sciences, Internal Medicine, Uppsala University, SE-751 85 Uppsala, Sweden; lars.lind@medsci.uu.se; 3 Department of Immunology, Genetics and Pathology, Uppsala University, SE-751 83 Uppsala, Sweden; cecilia.lindskog@igp.uu.se; 4 Division of Family Medicine and Primary Care, Department of Neurobiology, Care Sciences and Society, Karolinska Institute, SE-141 52 Huddinge, Sweden; johan.arnlov@ki.se

**Keywords:** nephropathy, biomarker, podocyte

## Abstract

**Background:** A previous report showed that the urine output of HPLBII-P in patients with diabetes mellitus and SARS-CoV-2 infection was increased as a sign of glomerular dysfunction. The aim of this report was to investigate the relation of the urine output of HPLBII-P to diabetes mellitus in two large community-based elderly populations, i.e., the ULSAM and PIVUS cohorts. **Methods:** HPLBII-P was measured by an ELISA in the urine of a community-based cohort of 839 men (ULSAM) collected at 77 years of age and in the urine of a community-based cohort of 75-year-old men, n = 387, and women, n = 401 (PIVUS). KIM-1, NGAL, and albumin were measured in urine and cathepsin S and cystatin C in serum. **Results:** HPLBII-P was significantly raised among males with diabetes in the ULSAM (*p* < 0.0001) and PIVUS cohorts (*p* ≤ 0.02), but not in the female cohort of PIVUS. In the female subpopulation of insulin-treated diabetes, HPLBII-P was raised (*p* = 0.02) as compared to women treated with oral antidiabetics only. In the ULSAM cohort, HPLBII-P was correlated to NGAL, KIM-1, and albumin in urine both in non-DM (all three biomarkers; *p* < 0.0001) and in DM (NGAL; *p* = 0.002, KIM-1; *p* = 0.02 and albumin; *p* = 0.01). Plasma glucose and HbA1c in blood showed correlations to U-HPLBII-P (r = 0.58, *p* < 0.001 and r = 0.42, *p* = 0.004, respectively). U-HPLBII-P and cathepsin S were correlated in the ULSAM group (r = 0.50, *p* < 0.001). No correlations were observed between U-HPLBII-P and serum creatinine or cystatin C. **Conclusions:** The urine measurement of HPLBII-P has the potential to become a novel and useful biomarker in the monitoring of glomerular activity in diabetes mellitus.

## 1. Introduction

The human phospholipase B precursor (HPLBII-P) was originally purified from white blood cells obtained from healthy human blood donors and was at that time only known as a hypothetical protein based on sequence analysis of the human genome [1]. Subsequently, we identified the nature of this protein since it displayed phospholipase activities with the ability to remove fatty acids from both sn-1 and sn-2 bonds in phospholipids, thus potentially giving rise to a broad panel of active fatty acid molecules when released from its cellular origin.

By means of our specific polyclonal antibodies, most human organ tissues were screened by immunohistochemistry for the expression of HPLBII-P [2]. Three organ tissues expressed the protein particularly well. These were neuronal cells, gastrointestinal and kidney tissues, in addition to bone marrow cells. By means of an ELISA, we measured HPLBII-P in gastrointestinal material and found interesting relations to GI diseases such as IBS [3] and IBD [2]. Our assay also allows for the measurement of the very low concentrations found in urine, and such measurements showed that in a cohort of COVID-19, this biomarker was closely related to acute kidney injury (AKI). We also observed in this cohort highly raised urine concentrations of HPLBII-P in a subgroup of patients with diabetes mellitus, which prompted us to investigate such a relationship further. For this purpose, we have measured HPLBII-P in urine in two community-based cohorts of elderly men and women, i.e., the ULSAM and PIVUS cohorts. The ULSAM study (the Uppsala Longitudinal Study of Adult Men) was initiated in 1969–1974 with a focus on the study of diabetes and cardiovascular disease [4]. The PIVUS cohort (The Prospective Investigation of the Vasculature in Uppsala Seniors (PIVUS) Study) was collected in the early 2000s and consisted of both males and females [5]. Our object was to compare the urine concentrations of HPLBII-P in these two cohorts to other currently used biomarkers of diabetes mellitus. The question that we raised was whether HPLBII-P might be a useful biomarker in urine for the management of diabetes mellitus.

## 2. Materials and Methods

Urine samples of the ULSAM cohort of men were collected at 77 years of age (n = 839). The collection was finalized in 2001 (sampling period 1998–2001). The urine samples of the PIVUS cohort were collected from people who were 75 years of age and contained 401 samples from women and 387 samples from men. All samples were stored in aliquots at −80 °C and had not been previously thawed and refrozen.

The healthy control group consisted of 45 women (median age 32 years, range 22–64 years) and 19 men (median age 26 years, range 22–63 years).

The studies were approved by the ethics committee of Uppsala University (ULSAM: Dnr97329, 14 October 1997 and PIVUS: Dnr 2005-M079, 2005) and the Declaration of Helsinki, and its subsequent revisions were followed.

The immunohistochemistry of kidney tissue was performed by the use of the rabbit polyclonal antibody raised against HPLBII-P. The immunohistochemistry procedure is documented in detail in the Human Protein Atlas (The Human Protein Atlas, www.proteinatlas.org).

HPLBII-P was measured by an ELISA (Diagnostics Development, Uppsala, Sweden). The measurement of cathepsin S (DY1183), NGAL (DY1757), and KIM-1 (DY1750) was performed by ELISA kits purchased from R&D systems (Minneapolis, MN, USA). All clinical data were blinded to the analysing personal. The analytical performances of the ELISAs were acceptable with CVs (Coefficients of Variation) in the range of 4–10% of duplicate samples.

Albumin, creatinine, and cystatin C were all measured in urine or serum/plasma as part of the clinical routine and performed by the clinical chemistry laboratory at University Hospital, Uppsala, Sweden.

## 3. Statistics

Non-parametric statistics was applied throughout unless otherwise indicated. For comparison between the independent results, the Mann–Whitney U-test was used, and for the comparison of the results of multiple groups, the Kruskal–Wallis ANOVA was used. Correlations between biomarkers were calculated by Spearman rank correlations. The statistical programme Medcalc was used in all calculations: MedCalc^®^ Statistical Software version 20.106 (MedCalc Software Ltd., Ostend, Belgium; https://www.medcalc.org).

## 4. Results

**Figure 1:**
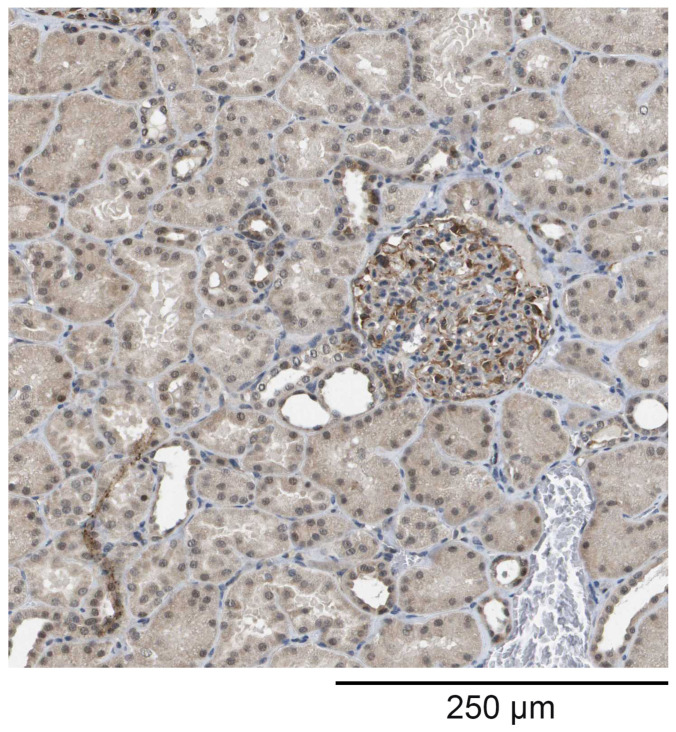
The staining of a nephron with the polyclonal anti-HPLBII-P antibodies. Most staining is seen in the glomeruli and probably associated with the podocytes, but also in some other structures such as the collecting ducts and tubuli.

The staining of kidney tissue with the polyclonal antibodies raised against HPLBII-P showed the distinct staining of podocytes in the glomeruli (Figure 1). Weak staining was also seen in some collecting ducts, but in few tubular cells.

**Figure 2:**
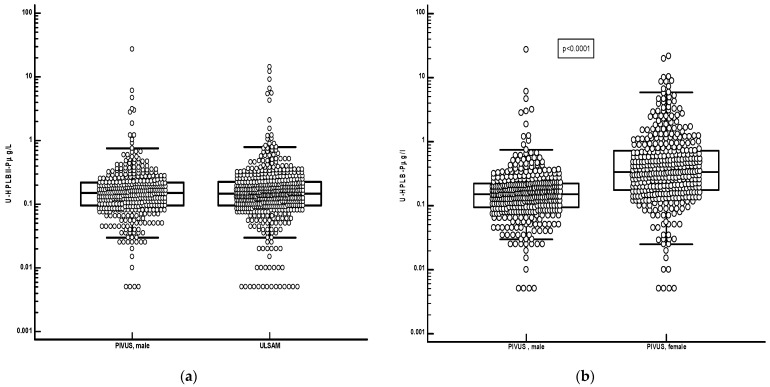
Panel (**a**) shows the urine concentrations of HPLBII-P in the male populations of the ULSAM and PIVUS cohorts. The urine concentrations were identical. Panel (**b**) shows the urine concentrations of HPLBII-P in males and females of the PIVUS cohort. The concentrations in the female population were significantly higher than in the male population (*p* < 0.0001, Mann–Whitney U-test).

**Figure 3:**
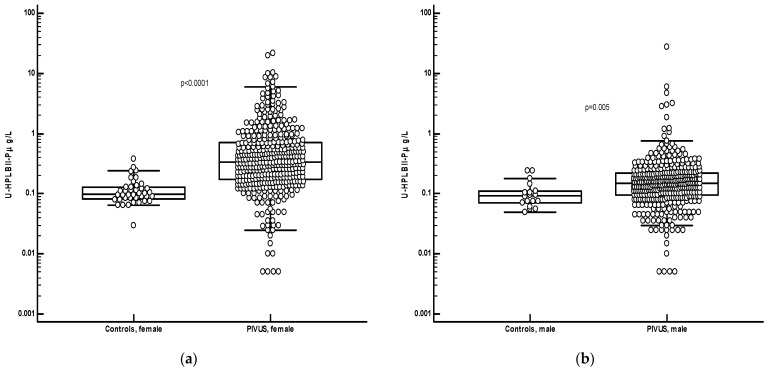
Panel (**a**) shows the comparisons of urine concentrations of the female population of the PIVUS cohort as compared to a healthy younger female population. The concentrations were significantly higher in the PIVUS cohort (*p* < 0.0001, Mann–Whitney u-test). Panel (**b**) shows the comparisons of the urine concentrations of the male population of the PIVUS cohort as compared to a healthy younger female population. The concentrations were significantly higher in the PIVUS cohort (*p* = 0.005, Mann–Whitney U-test).

**Figure 4:**
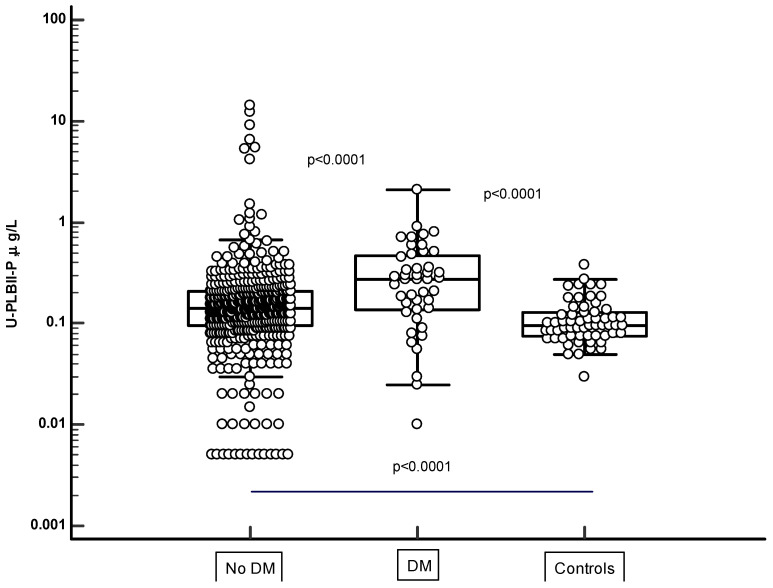
The urine concentrations of HPLBII-P in subjects with diabetes (BM) and no diabetes (no DM) in the ULSAM cohort. The statistical differences are indicated on the figure. Also shown are the differences between the healthy younger male population and the two ULSAM populations.

Figure 2 shows the distribution of HPLBII-P in the urine of the ULSAM and PIVUS cohorts. The concentrations of HPLBII-P were similar in the male ULSAM cohort and in the male sub cohort of PIVUS. A highly significant difference (*p* < 0.0001), however, was observed between males and females in the PIVUS cohort. It is shown in Figure 3 that the concentrations were significantly elevated in the PIVUS cohorts, both in males and in females, as compared to a group of younger healthy controls [females; age 35 years (95% CI 27–45) and males; age 27 years (95% CI 26–34)]. No difference between genders was seen in the control group. Also, the concentrations in the ULSAM cohort were significantly elevated as compared to the male subgroup of the healthy controls (*p* < 0.0001) (Figure 4). In the Supplementary File, we show the results in the PIVUS cohort after correction for urine creatinine concentrations. The difference between males and females became even larger after such a correction (*p* < 0.0001).

In Figure 4, the HPLBII-P concentrations in urine are shown in two populations of the ULSAM cohort, i.e., in those with no diagnosis of diabetes mellitus (n = 466) and in those with a diagnosis of diabetes mellitus (n = 44). The HPLBII-P concentrations were significantly higher in diabetes mellitus (*p* < 0.0001). In the PIVUS cohort, a significant elevation of the concentrations of HPLBII-P in diabetics was only seen among males (*p* = 0.019 and *p* = 0.012 after urine creatinine correction) but not in females (Table 1). Significantly higher concentrations were observed in subjects with diabetes treated with insulin as compared to those with no insulin treatment, either in comparison with the whole cohort without insulin (*p* = 0.026), or in comparison with the subjects who were treated with oral antidiabetics only (*p* = 0.028). A similar trend was seen in the ULSAM population, although the number of insulin-treated subjects was low, i.e., n = 3.

**Figure 5:**
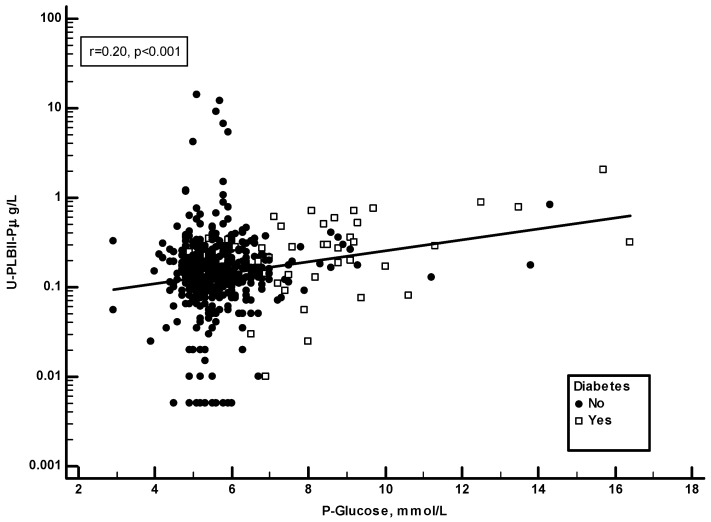
The correlation between plasma glucose concentrations and urine HPLBII-P concentrations in the ULSAM cohort (r = 0.20, *p* < 0.001). The results of the diabetic subjects are shown by the open squares.

**Figure 6:**
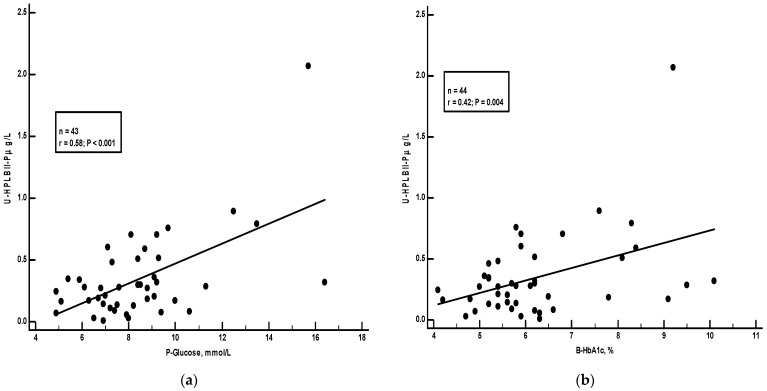
Panel (**a**) shows the correlation between plasma glucose concentrations and urine concentrations of HPLBII-P in the diabetic subpopulation of the ULSAM cohort. Panel (**b**) shows the correlation between blood HbA1c percentage and urine concentrations of HPLBII-P in the diabetic subpopulation of the ULSAM cohort. The statistics are given on the figure.

Plasma glucose and HbA1c were recorded in the ULSAM study. Figure 5 shows a significant positive correlation in the ULSAM cohort between HPLBII-P and plasma glucose (r = 0.20, *p* < 0.001). However, as suggested on the figure, this correlation was mainly due to the findings in those subjects with diabetes mellitus. By the calculation of correlations on the subgroup of diabetics separately, a strong correlation (r = 0.58, *p* < 0.001) was found to plasma glucose, but also to the proportion of HbA1c in blood (r = 0.42, *p* = 0.004) (Figure 6).

**Figure 7:**
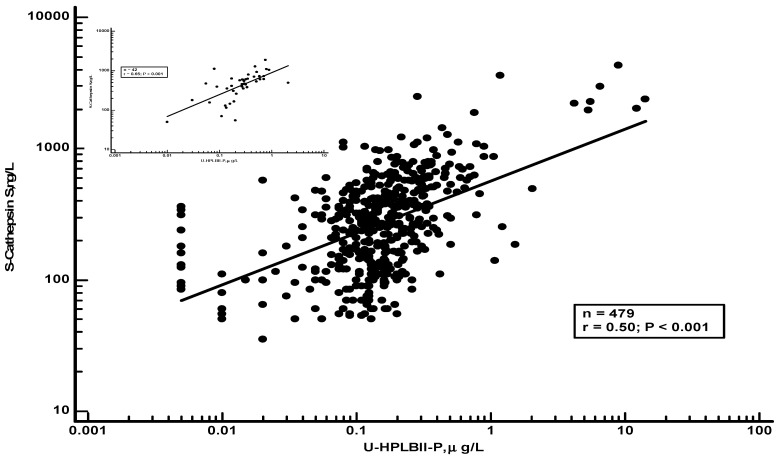
The correlation between serum concentrations of cathepsin S and urine HPLBII-P concentrations in the ULSAM cohort. The insert shows the results with the diabetic population of the ULSAM cohort. The statistics are given in the figures.

In Table 2, we show the results of the selected biomarkers and their correlations to HPLBII-P in urine from non-diabetics or diabetics. Significant correlations were found to the kidney tubular biomarkers NGAL and KIM-1 in urine, as well as to the glomerular biomarker albumin in urine. Significant correlations were observed between albumin in urine and HPLBII-P in urine both in males and females (Supplementary Figure S2). We also observed a significant correlation in males with diabetes in the PIVUS cohort (r = 0.30, *p* = 0.025, n = 61) but not in women. However, the strongest correlations were seen between cathepsin S in serum and the urine concentrations of HPLBII-P (Figure 7). After the correction for the creatinine concentrations in urine, similar results were obtained in the ULSAM cohort as without correction (Supplementary Table S1).

**Figure 8:**
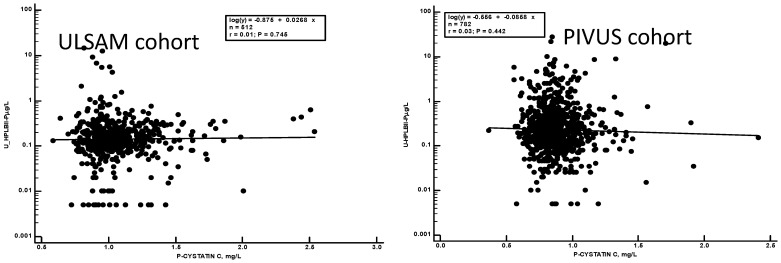
The correlations of the urine output of HPLBII-P and plasma concentrations of cystatin C. (**Left**) panel shows the results in the ULSAM cohort and the (**right**) panel the results in the PIVUS cohort. No correlations were apparent in the two cohorts.

No relations of the urine concentrations of HPLBII-P to filtration biomarkers such as serum creatinine or plasma cystatin C were observed in either patient cohort (Figure 8).

## 5. Discussion

The data in this report confirm our previous findings of HPLBII-P being closely related to the glomerular function of diabetes mellitus, as measured by albumin excretion, and to the tubular function, as measured by the close relationships to the tubular biomarkers KIM-1 and NGAL (submitted). The association of these biomarkers to diabetes has been investigated previously in numerous studies [6,7,8]. The relationships of these biomarkers to HPLBII-P were also true for the two community-based populations of females and males in the ULSAM and PIVUS cohorts. However, several other key findings were also obtained in the study of these two cohorts. One was the much higher overall urine concentrations of HPLBII-P in females as compared to males and with no significant differences between those with diabetes mellitus and non-diabetics. Another was the higher concentrations in both cohorts as compared to a healthy cohort of younger people. Thus, both gender and age seemed to determine the excretion of HPLBII-P. The third unexpected observation was the higher concentrations of HPLBII-P in urine in patients treated with insulin, which was most obvious in the females of the PIVUS cohort. A fourth intriguing observation was the very close correlation to the biomarker cathepsin S in serum. A negative finding was the absence of any relationship to kidney function, i.e., glomerular filtration, as measured by the serum/plasma concentrations of creatinine and cystatin C.

Given the fact that the presence of HPLBII-P is most likely a consequence of local processes in the kidney, the raised concentrations of this biomarker probably reflect the conditions of the glomeruli. One may be the structural changes and injury of the glomeruli and their podocytes as part of a nephrosclerotic process which is known to affect the majority of elderly people [9]. An alternative interpretation is the increased secretory activity of the podocytes, for which there is no support or evidence presently at hand for its interpretation. However, hyperglycaemia was shown to affect podocytes directly [10], and we indeed found significant correlations between HPLBII-P in urine and the plasma glucose concentration in this and in our previous report. Also, injury to the podocytes might result in increased concentrations of HPLBII-P in urine and increased podocyte mRNA [11], and nephrin and podocin [12,13,14,15,16] in the urine were suggested to reflect podocyte injury in patients with diabetes mellitus. Against the simple interpretation of the raised urine output of HPLBII-P being a consequence of aging is the fact that elderly women were found to have much higher concentrations in their urine than elderly males. Thus, the aging processes of the kidney were previously shown to affect sexes differently, with a faster decline in kidney function in males [17,18]. The higher output of HPLBII-P in women therefore remains unexplained. The elevated risk of patients with diabetes type 1 to develop organ failure such as kidney failure is well established [6] and may also be reflected in our findings by the highly elevated concentrations of HPLBII-P in urine in these patients. In this regard, we did not see any gender differences, although the number of patients was small.

In our earlier studies, we found that the serum biomarker cathepsin S was associated with the risk of developing diabetes, probably by impairing insulin sensitivity [19]. In this report, we found a remarkable association between cathepsin S and the urine output of HPLBII-P not only in the subgroup of patients with diabetes mellitus but also in the whole ULSAM cohort of elderly men. Cathepsin S is a macrophage-derived proteolytic enzyme and has been implicated in the destruction of glomerular structures in many renal diseases [20]. The close correlation with HPLBII-P, therefore, indicates that HPLBII-P may be an interesting biomarker in the monitoring of glomerular activity in diabetes mellitus and other renal diseases.

HPLBII-P is a fairly complicated molecule of 130 kD and made up of pairs of two subunits of approximately 22 and 42 kD, respectively. Theoretically, therefore, the significant correlation to filtration biomarkers such as albumin could reflect the filtration of such subunits since our polyclonal antibody-based ELISA cannot distinguish between the intact protein of 130 kD or the two subunits [1]. Whether these subunits exist in circulation is not known and quite difficult to determine given the fact that plasma concentrations are very low, i.e., below 1 μg/L. However, in preliminary results from plasma measurements, we do not find any signs of raised concentrations in plasma from patients with filtration disturbances, which would be expected with molecules of this size. Based on these facts, we strongly believe that the main portion of HPLBII-P in urine is derived from local production in the glomeruli and that the correlation to albumin in urine is mainly a parallel phenomenon reflecting kidney injury. However, the limitation of this study relates to the above, which is the uncertainty of whether HPLBII-P is produced locally by glomerular cells or is a mere reflection of destruction and injury to the glomeruli. Thus, a detailed study of the origin and mechanisms of the secretion of HPLBII-P is indeed warranted before we can safely conclude that this biomarker is the reflection of local processes in the glomeruli, possibly reflecting some unique process in the kidney.

We conclude from this study that the urine biomarker HPLBII-P is a sensitive reflection of glomerular activity in patients with diabetes mellitus, but also in non-diabetic aging populations. We hypothesize that HPLBII-P is a reflection of podocyte function, the function of which is a possible key to the understanding of diabetic nephropathy, as suggested by Reddy et al. several years ago [21], but also for the understanding of the glomerular filtering process in general [22]. This biomarker, therefore, has the potential to become a clinical tool in the monitoring of kidney function and in the prevention of the development of chronic kidney disease. Our findings merit further investigations of the potential clinical utility of HPLBII-P in urine.

## Figures and Tables

**Table 1:** Urine output of HPLBII-P in the PIVUS and ULSAM cohorts in relation to diabetes mellitus. Ns = Non-significant.

	Non-Diabetes μg/L, Median (95% CI) N	Diabetes μg/L, Median (95% CI) N	*p*-Value Mann–Whitney Non-Diabetes vs. Diabetes	Insulin, No μg/L, Median (95% CI) N	Insulin, Yes μg/L, Median (95% CI) N	*p*-Value Mann–Whitney Insulin No vs. Insulin Yes
**PIVUS, male**	0.145 (0.13–0.16) 324	0.173 (0.144–0.210) 62	*p* = 0.019	0.150 (0.135–0.160) 373	0.195 (0.127–0.320) 14	*p* = 0.13
**PIVUS, female**	0.335 (0.305–0.410) 355	0.370 (0.264–0.653) 46	Ns	0.335 (0.300–0.398) 389	1.29 (0.288–1.64) 13	*p* = 0.026
**ULSAM, male**	0.140 (0.132–0.153) 466	0.273 (0.185–0.320) 44	*p* < 0.0001	0.145 (0.135–0.160) 507	0.510 (0.210–0.705) 3	*p* = 0.024

**Table 2:** Biomarkers in urine and serum in the subpopulations of diabetes and non-diabetes in the ULSAM cohort and their correlations to HPLBII-P in urine. Ns = Non-significant.

Biomarker in Urine	Non-DM Median (95% CI)	DM Median (95% CI)	*p*-Value (Mann–Whitney)	Correlation to U-HPLBII-P Non-DM	Correlation to U-HPLBII-P DM
NGAL, μg/L	17.2 (15.7–18.3) N = 565	26.4 (14.0–34.1) N = 50	0.04	R_s_ = 0.25 *p* < 0.0001	R_s_ = 0.47 *p* = 0.002
KIM-1, ng/L	792 (730–873) N = 562	1066 (727–1290) N = 50	0.01	R_s_ = 0.19 *p* < 0.0001	R_s_ = 0.35 *p* = 0.02
Albumin, mg/L	7 (6–8) N = 636	15 (11.9–30) N = 60	0.0003	R_s_ = 0.253 *p* < 0.0001	R_s_ = 0.39 *p* = 0.01
**Biomarker in serum**					
Cystatin C, mg/L	1.03 (1.02–1.05) N = 702	1.12 (1.03–1.16) N = 69	0.02	R_s_ = 0.03 Ns	R_s_ = −0.19 Ns
Cathepsin S, ng/L	265 (245–290) N = 456	512 (399–605) N = 46	<0.0001	R_s_ = 0.42 *p* < 0.0001	R_s_ = 0.70 *p* < 0.0001

## Data Availability

The data of the study can be obtained from P.V. The data is not publicly available due to patent applications.

## Supplementary Materials

The following supporting information can be downloaded at: https://www.mdpi.com/article/10.3390/jcm13092629/s1, Figure S1: The U-PLBII-P in males and females after urine creatinine correction; Figure S2: The correlations between urine albumin and urine HPLBII-P corrected for urine creatinine in the PIVUS cohort as separated by gender; Table S1: Ulsam study. Biomarkers in urine corrected for Creatinine.
