# Supplementary material for: The Human Phospholipase B-II Precursor (HPLBII-P) in Urine as a Novel Biomarker of Increased Glomerular Production or Permeability in Diabetes Mellitus?
Source: J Clin Med. 2024 Apr 30;13(9):2629. doi: 10.3390/jcm13092629 (PMC11084184; doi:10.3390/jcm13092629)

Supplementary figure and table

All urine data were recalculated after correction with urine concentrations of Creatinine with minor impact on the overall results.

Supplementary Figure S1

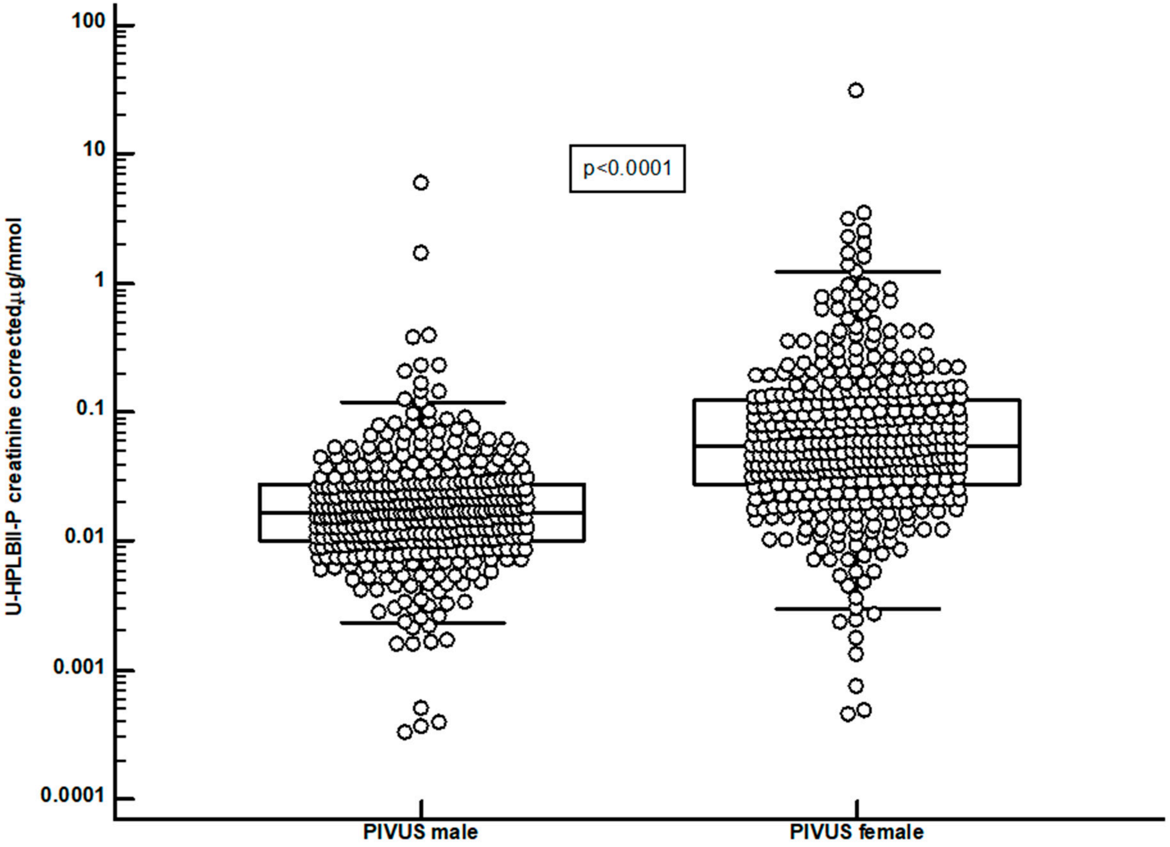

Supplementary Table S1

ULSAM study

Biomarkers in urine corrected for Creatinine

**Biomarker in urine**	**Non-DM Median (95% CI)**	**DM Median (95% CI)**	**P-value (Mann-Whitney)**	**Correlation to U-HPLBII-P/Crea Non-DM**	**Correlation to U-HPLBII-P/Crea DM**
NGAL/Crea μg/mmol	1.9 (1.8-2.1) N=565	2.3(1.8-3.8) N=50	0.01	R_s=0.35 p<0.0001	R_s=0.50 p=0.001
KIM-1/Crea ng/mmol	96 (88-102) N=562	140 (97-185) N=50	0.002	R_s=0.33 p<0.0001	R_s=0.49 p=0.001
Albumin/Crea mg/mmol	0.79 (0.70-0.91) N=635	1.86 (1.35-3.96) N=60	<0.0001	R_s=0.34 p<0.0001	R_s=0.37 p=0.01
**Biomarker in serum**					
Cystatin C, mg/L	1.03 (1.02-1.05) N=702	1.12 (1.03-1.16) N=69	0.02	R_s=0.05 Ns	R_s=-0.29 Ns
Cathepsin S, ng/L	265 (245-290) N=456	512 (399-605) N=46	<0.0001	R_s=0.33 p<0.0001	R_s=0.55 p=0.0002

Supplementary Figure S2: The correlations between urine albumin and urine HPLBII-P corrected for urine creatinine in the PIVUS cohort as separated by gender. The statistics are shown in the figure below.

PIVUS

Female

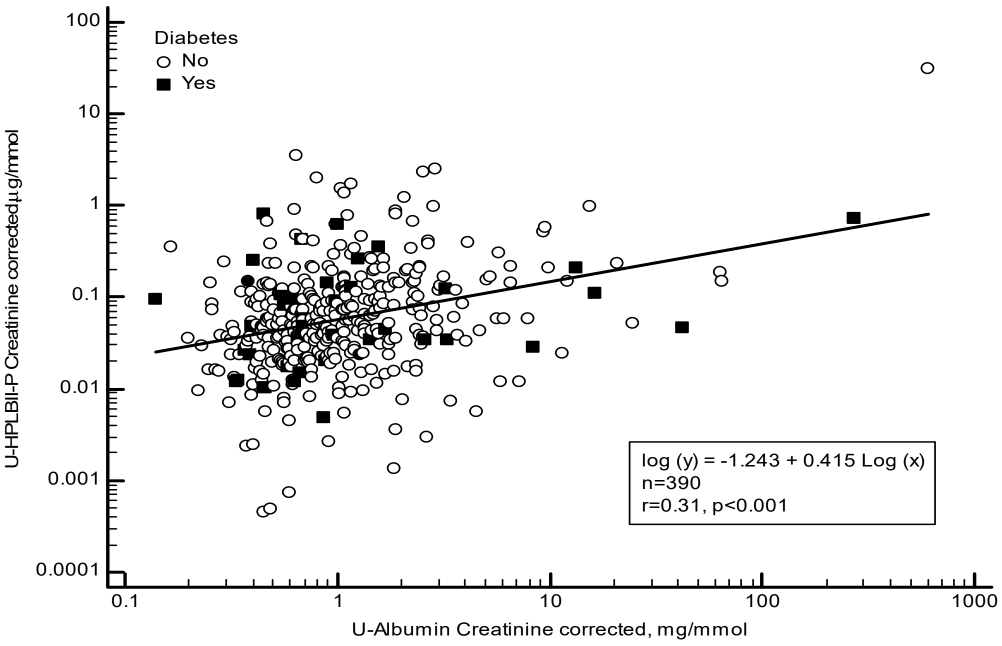

PIVUS

Male

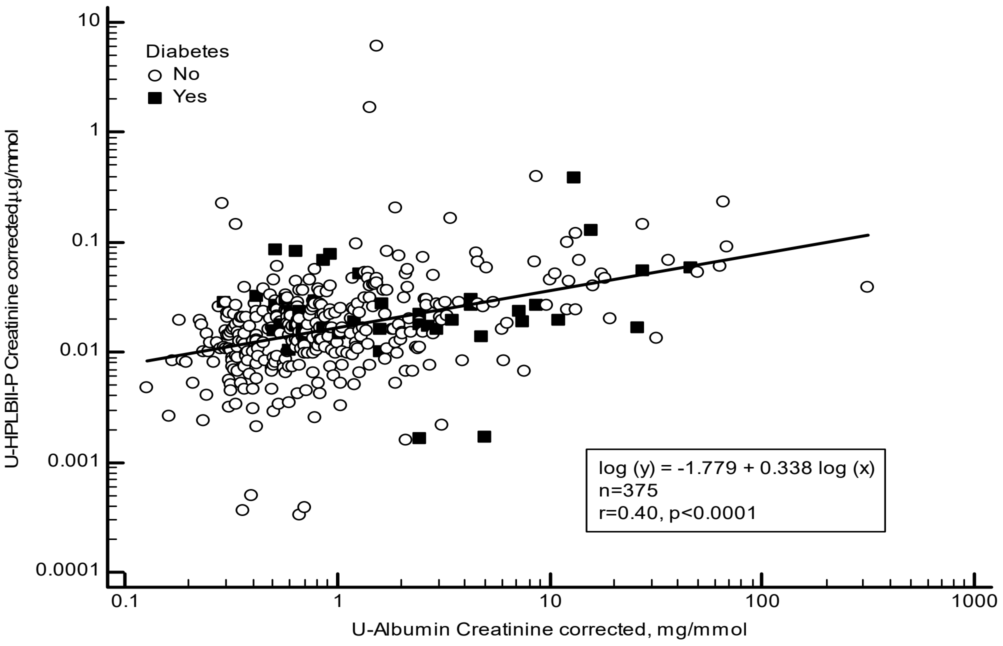

Supplement: Supplementary file 1 [file jcm-13-02629-s001.zip › jcm-2896280-supplementary.pdf]
